# Regulation of nitrogen fixation from free-living organisms in soil and leaf litter of two tropical forests of the Guiana shield

**DOI:** 10.1007/s11104-019-04012-1

**Published:** 2019-04-01

**Authors:** Leandro Van Langenhove, Thomas Depaepe, Sara Vicca, Joke van den Berge, Clement Stahl, Elodie Courtois, James Weedon, Ifigenia Urbina, Oriol Grau, Dolores Asensio, Josep Peñuelas, Pascal Boeckx, Andreas Richter, Dominique Van Der Straeten, Ivan A. Janssens

**Affiliations:** 1 Centre of Excellence PLECO (Plants and Ecosystems), Department of Biology, University of Antwerp, Wilrijk, Belgium; 2 Laboratory of Functional Plant Biology, Department of Biology, Ghent University, Ledeganckstraat 35, B-9000 Ghent, KL Belgium; 3 INRA, UMR Ecology of Guiana Forests (Ecofog), AgroParisTech, Cirad, CNRS, Université des Antilles, Université de Guyane, 97387 Kourou, French Guiana; 4 Laboratoire Ecologie, évolution, interactions des systèmes amazoniens (LEEISA), Université de Guyane, CNRS, IFREMER, French Guiana, 97300 Cayenne, France; 5 Department of Ecological Science, Vrije Universiteit Amsterdam, De Boelelaan 1085, 1081 HV Amsterdam, The Netherlands; 6 CSIC, Global Ecology Unit CREAF-CSIC-UAB, 08193 Bellaterra, Catalonia Spain; 7 CREAF, 08193 Cerdanyola del Vallès, Catalonia Spain; 8 Department of Applied Analytical and Physical Chemistry, Faculty of Bioscience Engineering, Isotope Bioscience Laboratory – ISOFYS, Ghent University, Coupure Links 653, 9000 Ghent, Belgium; 9 Department of Microbiology and Ecosystem Science, University of Vienna, Althanstr. 14, 1090 Vienna, Austria

**Keywords:** Free-living nitrogen fixation, Tropical forest, French Guiana, Nutrients, Phosphorus, Molybdenum

## Abstract

**Background and aims:**

Biological fixation of atmospheric nitrogen (N_2_) is the main pathway for introducing N into unmanaged ecosystems. While recent estimates suggest that free-living N fixation (FLNF) accounts for the majority of N fixed in mature tropical forests, the controls governing this process are not completely understood. The aim of this study was to quantify FLNF rates and determine its drivers in two tropical pristine forests of French Guiana.

**Methods:**

We used the acetylene reduction assay to measure FLNF rates at two sites, in two seasons and along three topographical positions, and used regression analyses to identify which edaphic explanatory variables, including carbon (C), nitrogen (N), phosphorus (P) and molybdenum (Mo) content, pH, water and available N and P, explained most of the variation in FLNF rates.

**Results:**

Overall, FLNF rates were lower than measured in tropical systems elsewhere. In soils seasonal variability was small and FLNF rates differed among topographies at only one site. Water, P and pH explained 24% of the variation. In leaf litter, FLNF rates differed seasonally, without site or topographical differences. Water, C, N and P explained 46% of the observed variation. We found no regulatory role of Mo at our sites.

**Conclusions:**

Rates of FLNF were low in primary rainforest on poor soils on the Guiana shield. Water was the most important rate-regulating factor and FLNF increased with increasing P, but decreased with increasing N. Our results support the general assumption that N fixation in tropical lowland forests is limited by P availability.

**Electronic supplementary material:**

The online version of this article (10.1007/s11104-019-04012-1) contains supplementary material, which is available to authorized users.

## Introduction

Nitrogen (N) availability is a limiting factor for plant growth in a wide range of terrestrial ecosystems worldwide (LeBauer and Treseder 2008) and restricts the amount of carbon (C) that can be assimilated and stored in the terrestrial biosphere (Hungate et al. 2003; Penuelas et al. 2013; Zaehle et al. 2015). Globally, biological N fixation is the most important natural pathway for introducing previously inert N - namely N_2_ gas -, into unmanaged ecosystems (Galloway et al. 1995; Vitousek et al. 2013). Nitrogen is fixed by microorganisms, known as diazotrophs, that can reduce gaseous N (N_2_) into ammonia. Diazotrophs are divided in two groups: symbiotic and free-living. Symbiotic N fixers are generally found in root nodules and live in a mutualistic relationship with higher plants that allocate C to the N fixers in exchange for N. Free-living N fixers are hetero- or autotrophic bacteria or archaea inhabiting water, soil, rocks and leaf litter (Dynarski and Houlton 2018). Global terrestrial N inputs from biological N fixation have been estimated at 60 Tg y^−1^ (Vitousek et al. 2013), and biome-level comparisons suggest that tropical rain forests may fix more N than any other unmanaged ecosystem (Galloway et al. 2004; Reed et al. 2011). Until recently, a large proportion of tropical N fixation was attributed to symbiotic organisms due to the high abundance of leguminous trees (Losos and Leigh 2004), typically associated with symbiotic N fixers. The relative importance of symbiotic N fixation, however, has been questioned because it is facultative (Menge et al. 2009) and may decline to near zero in mature tropical rainforests (Barron et al. 2011; Batterman et al. 2013; Sullivan et al. 2014). In this context, N fixation by free-living organisms is thus increasingly considered to be more important, with recent estimates from multiple rain forests suggesting that a substantial amount of N is indeed fixed by free-living diazotrophs (e. g. Reed et al. 2007, Cusack et al. 2009, Černá et al. 2009, Barron et al. 2009, Wurzburger et al. 2012, Matson et al. 2014).

Because of its relevance for ecosystem functioning, N fixation and its rate-controlling factors have been the focus of previous research. Nitrogen fixation is energetically expensive, requiring much ATP for both the reaction itself (Gutschick 1981) and for maintaining an oxygen-poor intracellular environment for nitrogenase, the enzyme responsible for N fixation (Robson and Postgate 1980). The oxygen-poor environment can be created, either by an upregulated respiration rate, or developing cellular structures that can limit the entry of oxygen (Robson and Postgate 1980). This regulation is needed because oxygen binds to nitrogenase and inactivates the enzyme. Free-living N fixation (FLNF) is thought to decrease as N availability increases in the environment (Hartwig 1998), because uptake of inorganic N is less costly than N fixation (Gutschick 1981; Menge et al. 2009)).

Low phosphorus (P) availability has been reported to limit FLNF in several tropical environments (e.g., Pearson and Vitousek 2002; Reed et al. 2007), an observation often attributed to the diazotrophs’s high P requirement. Nonetheless, studies of P addition to tropical forests have reported contradictory responses. Some reported that FLNF in soils and plant litter increased in response to P additions (Benner et al. 2007; Reed et al. 2007), while others found no effect (Pérez et al. 2008; Barron et al. 2009). Recent studies suggest that the stimulation of N fixation by P addition may be due to molybdenum (Mo) contamination of the commonly used superphosphate fertiliser (Barron et al. 2009; Wurzburger et al. 2012). Molybdenum is a rock-derived trace element required to produce the FeMo cofactor necessary for the functioning of the most common group of nitrogenases (Igarashi and Seefeldt 2003). Molybdenum is thus associated more with fundamental enzymatic requirements than with the high energy consumption of N fixation, but limitation of both Mo and P has been documented in some forests (Barron et al. 2009; Wurzburger et al. 2012; Reed et al. 2013).

Energy in the form of external organic carbon (C) is another likely factor regulating the activity of free-living N fixers. Like other heterotrophic microbes, diazotrophs rely on extracellular organic C for respiration, and C supply may be more limiting than nutrient availability in free-living fixers (Hofmockel and Schlesinger 2007), even though the C cost of FLNF is not well-quantified (Dynarski and Houlton 2018). Lastly, seasonal variation in soil moisture can play a large role in the regulation of N fixation rates, because the rates are positively correlated with moisture content (Roskoski 1980). Seasonal differences in N fixation have indeed been observed, but the direction of these seasonal changes differed among studies. Studies conducted at different tropical sites have reported both higher (cf Reed et al. 2007) and lower (cf Matson et al. 2014) rates in the wet season compared to the dry season with likely factors besides moisture, such as changes in oxygen supply, causing the discrepancy in results.

Lowland tropical forests found on highly weathered old soils are typically considered to be N-rich, because they can accumulate, recycle and export large quantities of N (Vitousek and Sanford 1986; Hedin et al. 2009; Brookshire et al. 2012). This open N cycle is also corroborated by enriched δ^15^N values of soils and plant tissues, due to high gaseous and leaching losses of depleted ^15^N sources (Amundson et al. 2003). Simultaneously, these lowland tropical forests have usually been described as P limited because of biogeochemical theory predicting that P limitation should be prevalent in old, strongly weathered soils (Walker and Syers 1976; Wardle et al. 2004). There is also a wealth of indirect evidence including high N availability (Brookshire et al. 2012), high N:P ratios in leaves (Vitousek 1984) and correlations between forest properties and soil fertility at continental scale (Quesada et al. 2012; ter Steege et al. 2006. Locally, however, nutrient availability in tropical landscapes can vary with topography, but the magnitude and direction of this variation is variable and influenced by, e.g., terrain steepness and rainfall, leading to differences in physical denudation rates and solute transportation (Porder et al. 2005; Weintraub et al. 2015). Differences in soil type and redox status along topographical gradients may also affect nutrient availability (Tiessen et al. 1994).

Here we present results from a study carried out in mature tropical rainforests of French Guiana, where we measured FLNF in both soil and leaf litter. The rolling hills of French Guianese tropical forests, part of the Guiana Shield and perched upon parent material that is amongst the oldest and most weathered in the world (Hammond 2005), are characterised as very poor in mineral nutrients (van Kekem et al. 1996), with topography inducing spatially heterogeneous nutrient availabilities. We studied three distinct topographical positions at two different forest sites in the wet and dry season, with the aim of maximizing the range in soil nutrients and moisture. Our goals were (I) to determine and compare rates of FLNF in soils and leaf litter to other tropical forest studies, (II) to evaluate whether or not N fixation rates differed between sites or between seasons, and (III) to identify which environmental factors (or combination thereof) best explained the spatial and seasonal variation in N fixation rates.

## Materials and methods

### Study sites

The study was conducted at two primary forest sites in French Guiana, in the research stations of Paracou and Nouragues. Paracou is situated 15 km from the coast (5°15’N, 52°55’W), while Nouragues is located inland (4°05’N, 52°41’W). Annual rainfall quantities at both sites were similar, with Paracou receiving an average of 3100 mm year^−1^ for the period 2004–2015 (Aguilos et al. 2018) and Nouragues receiving an average of 2990 mm year^−1^ (Bongers et al. 2001). Mean annual air temperature is near 26 °C for both sites (Gourlet-Fleury et al. 2004; Bongers et al. 2001). The French Guianese climate is characterized by a wet and a dry season due to the north/south movement of the Inter-Tropical Convergence Zone. The region receives heavy rains from December to July and a dry period, typically characterized by less than 100 mm rainfall month^−1^, from August to November (Aguilos et al. 2018).

Topography at both sites is undulating with maximum slopes of approximately 15°. The elevational difference between hill summits and valleys is 20–50 m over horizontal distances of 200–400 m. Soils at the Paracou site are schist soils with veins of pegmatite along the Bonidoro series, a Precambrian metamorphic formation (Epron et al. 2006). Soils at the Nouragues site are also derived from the same Bonidoro series, and consist of mainly Caraib gneiss (Bongers et al. 2001). This Precambrian geological substrate is particularly low in P content compared to the generally younger, nutrient-richer soils of western Amazonia (Hammond 2005; Grau et al. 2017) and therefore soils at both sites are classified as nutrient-poor Acrisols (FAO-ISRIC-ISSS 1998). Soils at Paracou range from loamy sand to sandy loam, while soils at Nouragues contain much more clay and span the range of sandy loam to silty clay according to the USDA texture classification chart (Fig. S1).

### Experimental design

To maximize the natural variation in soil nutrient availability the experimental plots encompassed a topographical gradient. At each forest site twelve plots were installed, distributed over four hillslopes, with each hillslope having three plots located at three topographical levels: (1) bottom, i.e. just above the creek running through the valley, (2) slope, i.e. the intermediate section of the elevation, and (3) top, i.e., where the slope evens out and becomes the hilltop. Each plot was of 20 × 20 m in size. In total, 24 plots spread over two sites and three topographical categories per site were studied. Distances between the plots were 10–100 m and in each plot five soil and litter samples were collected. Four samples were collected at 2 m distance from each corner and a fifth sample was taken in the middle of the plot. A total of 120 sampling points (2 study sites, 3 topographical categories per site, 4 plots per category and 5 samples per plot) were sampled for both soil and leaf litter in both the wet and dry season (240 samples in total). Courtois et al. (2018) have provided more detailed information on the experimental design.

### N fixation

Nitrogen fixation rates were determined using the acetylene reduction assay (Hardy et al. 1968). Samples were collected in May and September 2016, in the wet and dry season, respectively. Samples of leaf litter were collected manually from the soil surface and soil samples were collected with a 2-cm diameter corer to a depth of 5 cm after removing all litter from the surface.

All samples were placed in clear 100 ml borosilicate jars. The jars were sealed with rubber septa and 10 ml of air was replaced with 10 ml of acetylene gas (welding grade, Air Liquide) to create a 10% headspace concentration by volume. The samples were then incubated in situ at ambient forest light (no direct sunlight) and temperature for 18 h. Sample moisture was not manipulated in any way, but was determined after the incubation as the weight loss after oven drying at 70 °C during 48 h.

After incubation, a subsample from the sample headspace was injected into a pre-evacuated 12 ml borosilicate vial (Labco Limited, Ceredigion, UK) and shipped to Ghent, Belgium for analysis. Ethylene concentrations were measured using laser-based photo-acoustic spectroscopy (ETD-300, Sensor Sense, The Netherlands). Parallel acetylene blanks (no leaf litter or soil) were created to assess background levels of ethylene in the acetylene gas (1.5 + − 0.4 nl ethylene ml^−1^ acetylene gas), which were subtracted from the sample ethylene concentrations. Controls for ethylene production in the soil or litter in the absence of acetylene gas were also assayed and were consistently below the detection limit of 0.01 nl ethylene ml^−1^ air. Soil and leaf litter samples that over the incubation time produced ethylene concentrations below the detection limit (0.01 nl ethylene ml^−1^ air) were recorded as half of this value.

We converted the rate of ethylene production, expressed as nmol ethylene g^−1^ sample h^−1^, into N fixation rates, expressed as kg N fixed ha^−1^ y^−1^ using the densities of the soil (to a depth of 5 cm) or leaf litter, and the theoretical ratio of 3 mol ethylene produced per mole N fixed (Benner et al. 2007; Cusack et al. 2009; Matson et al. 2014; Pearson and Vitousek 2001; Reed et al. 2007). The latter being based upon the conclusion that reducing 3 mol of acetylene to ethylene is equivalent to the 6 electron transfer involved in the reduction of one mole of N_2_ to ammonium (Seitzinger and Garber 1987). We attempted to measure uptake of ^15^N through the pool dilution assay (Furnkranz et al. 2008) in a subset of soil samples to gauge the actual ratio of moles ethylene produced per mole N fixed, but we failed to do so due to a combination of low soil FLNF rates and high background levels of N in these environmental samples. Other authors have reported encountering similar issues and could not measure ^15^N uptake in soil (Matson et al. 2014) or leaf litter (Menge et al. 2009) samples. Soil samples for determining bulk density to a depth of 5 cm were taken with Kopeck rings. The samples were oven dried at 105 °C for 48 h and bulk density was calculated by dividing soil weight by Kopeck ring volume. Leaf litter was collected in a 0.5 m^2^ wooden frame at five locations per plot and once per season for determining litter density. The litter was dried at 105 °C for 48 h and the density was calculated by dividing weight by area (kg ha^−1^).

### Chemical analyses

Total C, N, P and Mo contents in the soil and litter were determined on the same samples that were used for acetylene reduction. Ethylene production was first measured (see N fixation), and afterwards samples were oven dried at 70 °C for two days and then ground in a ball mill (Retsch, Germany). Total C and N contents were determined by dry combustion with an elemental analyser (Flash 2000, Thermo Fisher Scientific, Germany). Total P (mg kg^−1^) and Mo (mg kg^−1^) contents were determined by the sequential digestion of the ground soil and litter samples in heated strong acid (69% HNO_3_ and 30% H_2_O_2_), followed by analysis on an iCAP 6300 Duo ICP optical emission spectrometer (Thermo Fisher Scientific, Germany).

Soil texture and nutrient availability were determined on a composite sample made of three soil cores per sampling point, each core to a depth of 15 cm. Texture was determined on fresh soil using the hydrometric method (Gee and Bauder 1986). Soil particles were dispersed with sodium metaphosphate and the amounts of sand, silt and clay were determined using a hydrometer. Soil samples for measuring nutrient availability were collected in May and September 2015, sieved (2 mm) and split into three subsamples. The first subsample was extracted with 1 M KCl in a 1:2.5 w:v ratio for pH measurement and determination of available N. On this extract pH_KCl_ was measured with a pH meter (HI 2210–01, Hanna Instruments, USA), after which the extract was filtered through a 42 μm filter and the filtrate’s concentration of NH_4_^+^ and NO_3_^−^ was determined colorimetrically (SAN++ continuous flow analyser, Skalar Inc., The Netherlands). Available N (mg kg^−1^) was defined as the sum of the NH_4_^+^ and NO_3_^−^ concentrations. The second subsample was used to determine the gravimetric water content by measuring weight loss after drying at 105 °C during 48 h. The third subsample was used to determine extractable P and Mo. Soils were oven dried at 60 °C for 48 h after which extractable P was determined with Bray-P acid fluoride extraction (Bray and Kurtz 1945). Available Mo was determined through resin extraction (Wurzburger et al. 2012). The samples were mixed with water in a 1:6 ratio and five 2 cm^2^ strips of anion-exchange membrane (VWR Chemicals, USA) were added. This mixture was stirred for 24 h and the strips were then rinsed and eluted with 10% HNO3. Available P and Mo contents were determined with a iCAP 6300 Duo ICP optical emission spectrometer (Thermo Fisher Scientific, Germany).

### Literature comparison

To compare the FLNF rate results we found to those of other authors we performed a database search similar to the search carried out by Dynarski and Houlton (2018). We searched Web of Science using the keywords nitrogen, free-living, asymbiotic, fixation, soil, leaf litter and tropical forest. From the resulting studies we selected those that were performed in natural terrestrial tropical ecosystems. For studies that presented results from multiple time points or seasons, we averaged the data over the course of the study period. Studies that did not report any measure of variance were assigned a standard error of ¼ of the mean. Reported rates of FLNF were converted to nmol ethylene produced g^−1^ dry substrate h^−1^ in order to compare N fixation rates between studies. When N fixation rates were presented on a per area basis we used the bulk density of the N fixation substrate (soil or leaf litter) and the ethylene: N_2_ conversion factor indicated in the study to convert to nmol ethylene produced g^−1^ dry substrate h^−1^. When no conversion factor was indicated, we assumed the standard 3: 1 conversion factor (Hardy et al. 1968). Results of this database search are summarised in supplementary information Table S4.

### Data analysis

To assess the differences in N fixation rates and soil and leaf litter variables between sites and seasons we used linear mixed effects regression models (LMER), analysing soil and leaf litter data separately. We used site (Paracou or Nouragues), season (Wet or Dry) and topographical position (Bottom, Slope or Top) as fixed factors and plot identity as a random factor. The validity of the linear models’ assumptions (linearity, normality of residuals, no influential outliers, homoscedasticity) were evaluated with standard functions of R (R core team 2017, version 3.4.3), including diagnostic plots. Prior to analysis, data were log transformed if their distribution was right-skewed to improve normality of model residuals. Multiple comparisons within a factor were analysed using Tukey post hoc tests. We performed principal component analyses to visualize the correlations of previously standardised soil and leaf litter variables according to site, season and, if necessary, topography. We observed that a large proportion of our measured samples yielded ethylene production rates that fell below the detection limit (25% of samples in soil). To investigate if there were site-specific or seasonal patterns in the occurrence of values below detection limit, we transformed our ethylene production rates into binomial data (1 for measured rate and 0 for below detection limit rate) and analysed the resulting data with binomial generalized linear model (GLM) with season and site as factors.

To identify which set of physico-chemical variables significantly contributed to the observed variation in N fixation rates we performed a forward stepwise model selection, i.e. starting from a null model and retaining the predictor variable that led to the largest decrease in the Akaike information criterion (AIC), corrected for sample size (AICc). This process was iterated until no additional predictor reduced the model AICc by at least two units. This procedure was performed for the dataset as a whole, as well as for each combination of site and season that was shown to be significantly different in the mixed-effects models (see above) in soil and in leaf litter. For these analyses the measurements of FLNF that were below the detection limit were excluded to assess which predictor variables participate in regulating the FLNF that were detectable and thus participate to the ecosystem scale N fixation. The potential predictor variables for both leaf litter and soil were standardised to a mean = 0 and standard deviation = 1 prior to the model fitting procedure to avoid potential issues in interpretation and numerical stability due to differences in magnitude between variables. Potential predictor variables were gravimetric water content, C:N ratio, N:P ratio, total carbon, total nitrogen, total phosphorus and total molybdenum. For soil we additionally included available nitrogen, phosphorus and molybdenum, soil pH and soil texture. We present the best-fit model for each data subset, based on this forward stepwise procedure.

As an additional check of robustness we used an Akaike weight approach to assess the importance of predictor variables. We summed Akaike weights computed for all possible first-order models containing a given predictor, to obtain a measure of the relative variable importance for this predictor (Burnham and Anderson 2002). We did this for all abovementioned predictors. This approach yielded very similar results to the forward stepwise model selection, confirming the robustness of our analysis, but for readability reasons are not presented nor discussed in this paper. These results are, however, shown in supplements (Figs. S2 and S3). All analyses were carried out with the software package R (version 3.4.3) using packages lme4 (Bates et al. 2015), MASS (Venables and Ripley 2002), ggfortify (Tang et al. 2016) and AICcmodavg (Mazerolle 2017).

## Results

Rates of FLNF were on average 0.015 ± 0.003 (standard error) nmol ethylene g^−1^ h^−1^ or 0.57 ± 0.10 kg N ha^−1^ y^−1^ in soil and 0.25 ± 0.04 nmol ethylene g^−1^ h^−1^ or 0.09 ± 0.01 kg N ha^−1^ y^−1^ in leaf litter. Per unit mass, FLNF rates were thus, on average, tenfold higher in leaf litter than in the top 5 cm of the soil (Table 1). However, when reported per unit area, FLNF rates were lower in litter than soil, due to the huge difference in density between the sample types (Table 1).

**Table 1:** Range, median, mean and standard error (SE) of FLNF rates at forest sites Paracou and Nouragues

			Paracou						Nouragues					
			Wet			Dry			Wet			Dry		
			Bottom	Slope	Top	Bottom	Slope	Top	Bottom	Slope	Top	Bottom	Slope	Top
Soil	Ethylene production (nmol g^−1^ h^−1^)	Minimum	BDL*	BDL	BDL	BDL	BDL	BDL	BDL	BDL	BDL	BDL	BDL	BDL
		Median	0.006	0.005	BDL	0.015	0.008	0.001	0.002	0.003	0.004	0.014	0.006	0.012
		Mean	0.012	0.017	0.002	0.014	0.013	0.003	0.003	0.005	0.028	0.055	0.010	0.037
		Maximum	0.044	0.191	0.022	0.029	0.056	0.018	0.006	0.034	0.404	0.298	0.086	0.460
		SE	0.003	0.009	0.001	0.002	0.002	0.001	0.000	0.002	0.020	0.030	0.004	0.021
	N fixation rate (kg N ha^−1^ y^−1^)	Minimum	BDL	BDL	BDL	BDL	0.001	BDL	0.020	BDL	BDL	0.001	BDL	BDL
		Median	0.296	0.183	0.008	0.738	0.350	0.060	0.084	0.119	0.129	0.537	0.228	0.395
		Mean	0.565	0.724	0.091	0.663	0.528	0.149	0.100	0.176	0.742	1.301	0.405	1.279
		Maximum	2.082	7.836	1.215	1.449	2.168	0.858	0.181	1.157	8.944	10.513	3.635	15.850
		SE	0.168	0.379	0.060	0.077	0.099	0.054	0.010	0.055	0.460	1.073	0.171	0.741
		Sign.	a	a	b	a	a	b	a	a	a	a	a	a
Litter	Ethylene production (nmol g^−1^ h^−1^)	Minimum	0.026	0.025	BDL	BDL	BDL	BDL	0.014	0.010	0.003	BDL	BDL	BDL
		Median	0.186	0.143	0.112	0.111	0.053	0.000	0.109	0.059	0.030	0.087	0.045	0.043
		Mean	0.524	0.433	0.403	0.376	0.290	0.001	0.346	0.300	0.150	0.207	0.046	0.139
		Maximum	3.018	2.500	3.976	2.981	4.554	0.007	2.898	2.407	1.252	0.930	0.120	2.112
		SE	0.188	0.127	0.186	0.156	0.219	0.001	0.145	0.119	0.063	0.056	0.008	0.103
	N fixation rate (kg N ha^−1^ y^−1^)	Minimum	0.011	0.005	BDL	BDL	BDL	BDL	0.005	0.003	0.001	BDL	BDL	BDL
		Median	0.052	0.045	0.044	0.028	0.015	0.000	0.042	0.017	0.008	0.043	0.015	0.019
		Mean	0.166	0.127	0.142	0.109	0.102	0.000	0.126	0.112	0.045	0.111	0.017	0.065
		Maximum	0.889	0.580	1.332	0.898	1.707	0.002	0.615	0.979	0.418	0.800	0.053	0.867
		SE	0.054	0.034	0.062	0.046	0.083	0.000	0.044	0.048	0.021	0.041	0.002	0.042
		Sign.	a	a	ab	b	b	c	a	abd	ac	bcd	ce	be

Rates measured in sample types soil and leaf litter are given for the wet and dry season separately and split up by landscape unit. Rates are expressed both on a mass basis as ethylene production rates and on an area basis as N fixation rates. Significant differences (*P* < 0.05) within a single site and sample type are indicated by differing letters. Calculation of median, mean and standard error and statistical analyses were performed from data including BDL* measurements. *BDL, below detection limit of 0.01 nl ethylene produced per ml air. This equates to a detection limit of 1*10^−4^ nmol g^−1^ h^−1^ and 7*10^−4^ kg N ha^−^

### Soil – N fixation

**Fig. 1:**
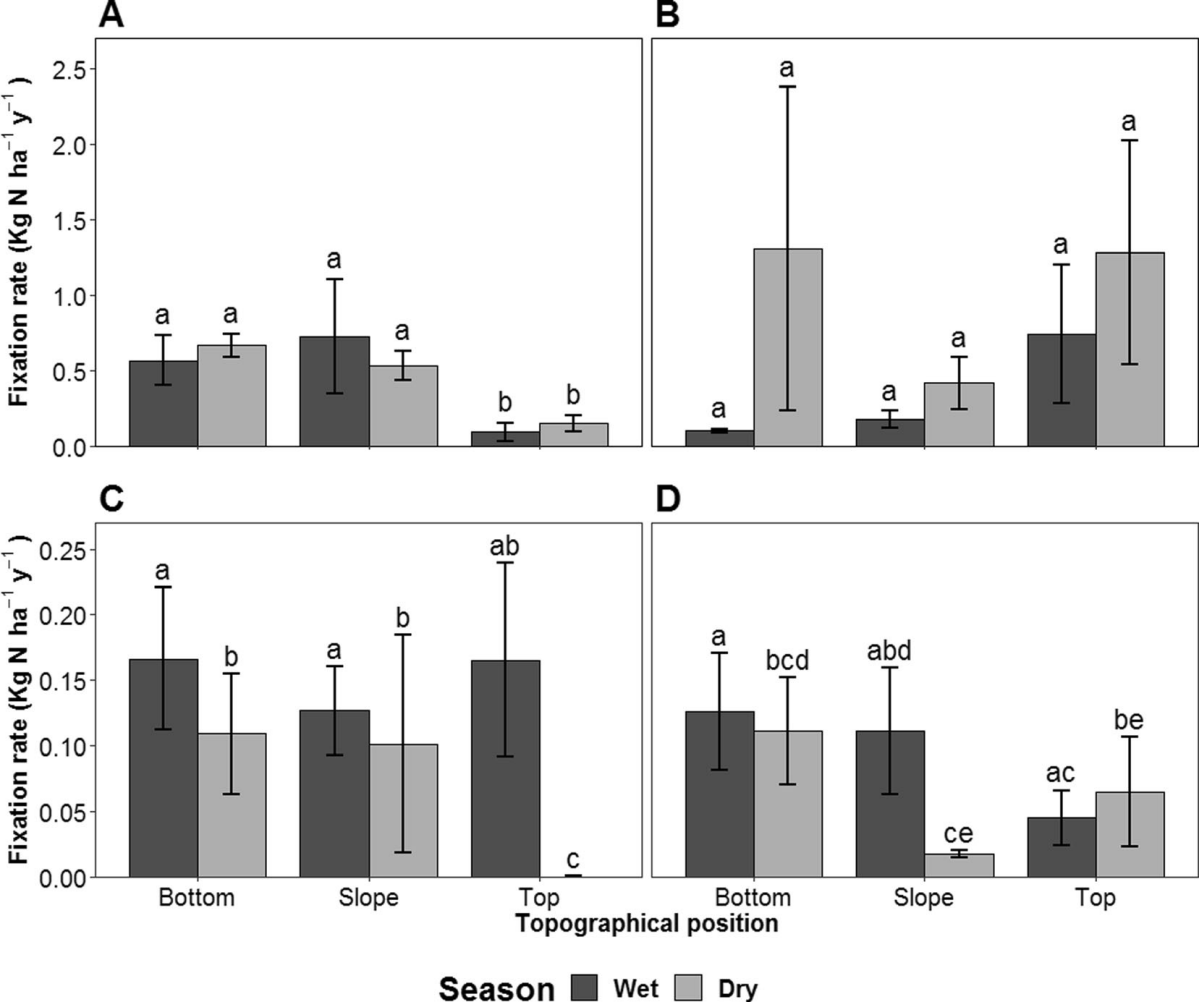
Area-based N fixation rates for soil at Paracou (**a**) and Nouragues (**b**) and for leaf litter at Paracou (**c**) and Nouragues (**d**). Rates represent the means (±1 SE) of the samples collected from the bottom, slope and top landscape positions in the wet (May) and dry (September) seasons (*N* = 20). Letters denote significant differences amongst different seasons and landscape units within a single site for soil or leaf litter at the P < 0.05 level. Significance testing was performed by mixed effects regression models using the log transformed values as measured values were non-normal. Data include measurements that were below the detection limit

Overall, soil FLNF rates did not differ between seasons (*P* = 0.27). The effect of topography on soil FLNF rates differed between Paracou and Nouragues (Site x Topography interactive effect; *P* = 0.021) (Fig. 1a, b). In Paracou, soil FLNF rates were 20 times higher (+/-SE = 6 to 65 times higher) in the bottom and slope plots than in the top plots (*P* = 0.047 and *P* = 0.009, respectively), but did not differ significantly between the bottom and slope plots. In Nouragues no differences were observed in the FLNF rates of the bottom, slope or top plots. A considerable proportion of the soil FLNF rates were below the detection limit (25%, out of 230 samples). Although the LMER did not reveal a significant effect of season on the soil FLNF rates, season did affect the number of samples whose FLNF rates were below the detection limit (Fig. S4 A & B), albeit differently in Paracou than in Nouragues (Site x Season interactive effect *P* = 0.004). Whereas in Paracou the number of soil FLNF rates that were below the detection limit did not differ between wet and dry season, in Nouragues values below the detection limit occurred primarily in the dry season (18 out of 52 samples versus 2 out of 56 samples, in the dry and wet season, respectively, *P* < 0.001). When conducting the LMER analysis on a subset of the Nouragues soil FLNF rates, excluding samples where FLNF rates were below the detection limit, we identified an effect of season (*P* = 0.008), with the highest rates occurring in the dry season. (Fig. S5 B).

### Soil – Environmental variables

**Fig. 2:**
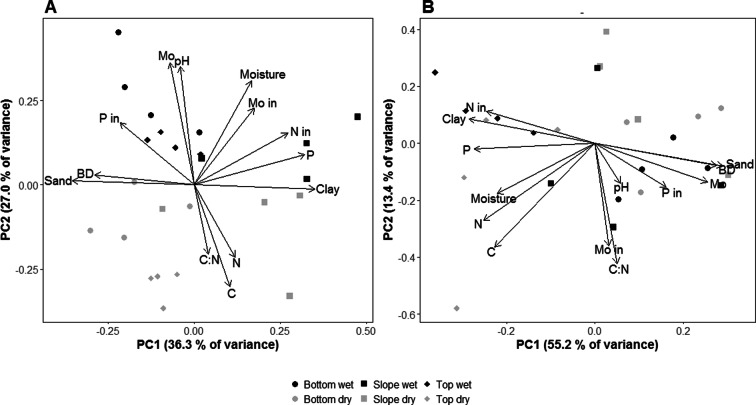
Principal Component Analysis of soil variables in Paracou (**a**) and Nouragues (**b**). Points on the graph are plot averages. C = total C, N = total N, P = total P, Mo = total Mo, C:N = C:N ratio, P in = available P, N in = available N, Mo in = available Mo, Moisture = water content, pH = pH, Clay = % clay, Sand = % sand and BD = bulk density

As each site had different soil FLNF rates relating to their topography, we performed a principal component analysis (PCA) for each site separately, allowing us to visualize site specific relationships among soil parameters. In Paracou, PC1 and PC2 explained 63.3% of the variation, with PC1 explaining 36.3% of the variation and containing most of the topographically induced variation (Fig. 2a). Clay content correlated with total P and available N and was highest on the slopes. PC2, explaining 27% of the variation, contained the seasonally-induced variation (Fig. 2a). Total C correlated with total N and was higher in the dry season, while total Mo correlated well with pH and was higher in the wet season. Available Mo correlated well with moisture and was also higher in the wet season. Available P was not heavily affected by season, but was higher in the bottom plots than on either the slopes or tops. In Nouragues PC1 and PC2 together explained 68.6% of the variation (Fig. 2b). The first principal component (PC1) explained 55.2% of the variation and, just as in Paracou, mostly contained topographically-induced variation. In Nouragues soil properties for top plots were clearly different from bottom and slope plots. Clay content correlated with total P and available N and all were highest in the top plots. In contrast to Paracou, however, total Mo correlated with available P and both were higher on the bottom and slope plots than on the top plots. Moisture, total C and total N correlated well with each other and clustered together at a ± 45° angle from PC1. Available Mo and C:N were correlated along PC2, which explained 13.4% of the variation. Lastly, pH also varied topographically and was highest on the bottom and slope plots, yet the variation was small. For all Pearson correlations, see Table S3 A and B.

### Leaf litter – N fixation

Leaf litter N fixation rates were not different between sites. On average, litter FLNF rates in the wet season were 7.5 to 13.5 times (± SE) higher (*P* < 0.001) than in the dry season. This difference between both seasons was further emphasized by the distinct seasonal difference in the proportion of FLNF measurements that were below the detection limit (Fig. S4 C & D): the vast majority (97%) of the 16% (33 out of 235) of litter FLNF measurements that were below the detection limit were from the dry season (binomial GLM, P < 0.001). Nonetheless, the impact of season interacted with that of topographical position (Season x Topography interactive effect P < 0.001) (Fig. 1c, d). In the wet season FLNF rates were similar among topographic positions, but in the dry season, rates on the top plots were 2 to 7.5 times (± SE) lower (*P* < 0.05) than rates on the bottom or slope plots, which did not differ from each other.

### Leaf litter – Environmental variables

**Fig. 3:**
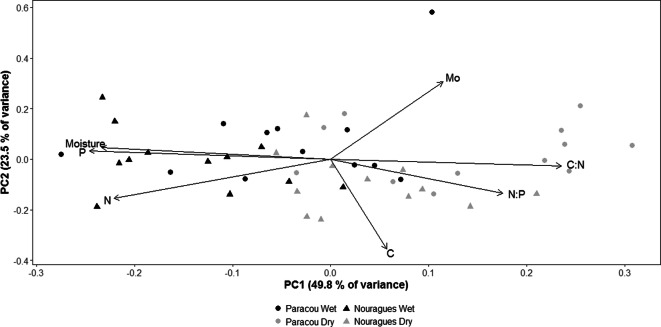
Principal Component Analysis of leaf litter variables in both Paracou and Nouragues and for both wet and dry season. Dots on the graph are averaged for plot. C = total C, N = total N, P = total P, Mo = total Mo, C:N = C:N ratio, N:P = N:P ratio and Moisture = water content

Because the LMER stated that FLNF rates were similar between sites we analysed the leaf litter stoichiometry of both sites together. Together, the first two principal components of the PCA accounted for 73.3% of the variation (Fig. 3). PC1 explained 49.8% of the variation and distinguished between the wet and dry season. Along this component, moisture, N and P content all correlated positively with each other and negatively with C:N and N:P ratio, indicating that moisture, N and P content was higher in the wet season, while C:N and N:P ratios were higher in the dry season. This was confirmed by linear effects regression analysis (Table S2). PC2, accounting for 23.5% of the variation, correlated positively with C content. The absence of a site effect on PC1 and PC2 indicates that leaf litter chemistry was similar for both sites. For all Pearson correlations, see Table S3 C.

### Drivers of FLNF in soils and litter

In soils, stepwise regression analyses indicated that soil water content, available P and pH were the primary drivers of N fixation rates (Table 2a), explaining 24% of the variation in soil FLNF rates. Because soil FLNF rates differed significantly between both sites, we ran the stepwise regression analysis again for each site separately. In Paracou, P exerted a strong effect, as both total P and available P had a positive influence on FLNF rates. Available N was negatively related to FLNF, and together with P, explained 36% of the variation. For Nouragues a model containing only total N, total C or water content explained 40% of the variation in FLNF rates. These three parameters were strongly correlated and the regression analysis deemed the models containing either one of them singularly equally valid (Table 2a).

**Table 2:** Results of the forward stepwise selection analyses for N fixation rates in soil (A) and leaf litter (B). For these analyses we excluded the measurements that were below the detection limit

A						
Site	Season	Parameters				R^2^
Par & Nou	Wet & dry	+0.57 Water* F_1,169_ = 15.6	+0.20 Available P* F_1,179_ = 4.8	+0.17 pH* F_1,180_ = 4.5		0.240
Par	Wet & dry	+1.1 Total P*** F_1,88_ = 44.7	+0.34 Available P** F_1,91_ = 7.2	−0.51 Available N** F_1,68_ = 9.3		0.362
		+0.25 Total N* F_1,88_ = 5.0				0.398
Nou	Wet & dry	+0.25 Total C* F_1,88_ = 4.7				0.396
		+0.25 Water* F_1,88_ = 5.0				0.401
B						
Site	Season	Parameters				
Par & Nou	Wet & Dry	+0.76 Water*** F_1,40_ = 24.67	−0.32 N:P** F_1,195_ = 8.34			0.457
Par & Nou	Wet	+1.71 Water** F_1,65_ = 9.10	+0.65 Total P** F_1,117_ = 10.69	+1.38 Total C** F_1,117_ = 8.64	+ 0.81 CN* F_1,115_ = 5.87	0.328
Par & Nou	Dry	+2.63 Water*** F_1,82_ = 26.26	−0.79 Total N* F_1,73_ = 6.70			0.676

Each row lists the parameters that were included into the model that provided the best fit, based on AICc values. Columns one and two give information regarding the dataset that was used. For soils (A) a model including data from both sites (Par, Paracou; Nou, Nouragues) and seasons was made along with models for each site separately because N fixation rates were different between sites. For leaf litter (B) a model including data from both sites and seasons was made along with models for each season separately because seasonality affected N fixation. Parameters that had a significant effect (* = p < 0.05, ** = p < 0.01 and *** = p < 0.001) on N fixation rates are given with their F values. Plus or minus numbers represent beta values. R^2^ was calculated for the fit of the modelled and measured fixation rates

^1^The stepwise selection identified total N, total C and water content as equally important for determining FLNF rates in Nouragues soils. This is because these three variables are highly correlated and behave similarly in the model. Explanations as to why this occurs are offered in the text

In litter, across both sites and seasons, about 46% of the variation in FLNF rates was explained by water and N:P ratio (Table 2b). While water had a positive effect on FLNF rates, N:P ratio showed a negative effect. As FLNF rates differed between seasons we ran the stepwise analysis again for each season separately. In the wet season 33% of the variation could be explained by water, C content, P content and C:N ratio. The model for the dry season explained about 68% of the variation and was dependent on the positive influence of water and the negative influence of N content.

## Discussion

Overall, the sum of FLNF rates of soils and leaf litter measured in this study fall into the lower end of the 0.1–60 kg N ha^−1^ y^−1^ range reported for FLNF on tropical forest floors worldwide (Reed et al. 2011), and much below the more recent estimate of 15–36 kg N ha^−1^ y^−1^ fixed in tropical forests (Pajares and Bohannan 2016) and the average ethylene production rate of 5.32 nmol ethylene g^−1^ h^−1^ reported in a recent meta-analysis on N fixation rates in tropical forest ecosystems (Dynarski and Houlton 2018). The FLNF rates found in the present study are much lower than those found in Costa Rica (Reed et al. 2007; Reed et al. 2010; Reed et al. 2013), Panama (Barron et al. 2009; Wurzburger et al. 2012), Puerto Rico (Cusack et al. 2009) or in the younger sites along a Hawaiian chronosequence (Crews et al. 2000). An overview of FLNF rates reported by these authors can be found in Table S4. Sullivan et al. (2014) measured N fixation rates in Costa Rica, about 35 km away from where Reed et al. (2007) conducted their study. These authors reported soil and leaf litter FLNF rates that were lower than those previously published by Reed et al. (2007), but while their reported soil FLNF rates were similar to those found in our study, their reported leaf litter FLNF rates were still twice as high as the rates we found. Other studies of FLNF rates conducted in less fertile tropical forests, e.g. on the higher altitudes of an altitudinal transect in Ecuador (Matson et al. 2014) or in the older sites of a Hawaiian chronosequence (Crews et al. 2000), reported FLNF rates comparable to those found in the present study. In Hawaii, Crews et al. (2000) measured decreasing rates of leaf litter FLNF on increasingly older soils and attributed this decrease to a combination of lower concentrations of geologically cycled nutrients, such as P, and high N pools at their oldest sites. This, coupled with increases in FLNF rates after P fertilization, led them to one of their main conclusions; that low P availability limited FLNF rates at their older sites. It would, however, be inaccurate to conclude that soil P alone determines N fixation since, for example, in Panama (Wurzburger et al. 2012) two of the studied sites along a total soil P gradient containing high (AVA) and medium (Gigante) levels of total soil P displayed the lowest rates of FLNF in their study.

### Soil free-living N fixation

At both sites moisture was important in the regulation of FLNF (Table 2a). Water is essential for all microbes, but for diazotrophs in particular water plays an important role in regulating activity. Not only does their metabolism require sufficient amounts of water, but nitrogenase activity is hindered in the presence of oxygen (O_2_) (Hill 1988). Oxygen binds to nitrogenase and inhibits its function (Hartmann and Burris 1987) and because increased soil moisture decreases soil O_2_ concentrations, water content can have a large impact on soil FLNF rates. As diazotrophs are mainly heterotrophic the thickness of the soil water film, which is important for the diffusion rates of extracellular enzymes and soluble organic-C substrates and is directly affected by soil water content (Davidson and Janssens 2006), will also play a role.

In Paracou, we observed no seasonal effect on soil FLNF rates and although rates were typically reported to be higher in the wet season than in the dry season (e. g. Hofmockel and Schlesinger 2007; Reed et al. 2007), Matson et al. (2014) have shown that the opposite can also occur. They postulated that it is likely that moisture fluctuations were not directly responsible for their observed seasonal changes in FLNF rates, just as in our study it might not have contributed to unchanging rates. Their reasoning was based on the fact that cyanobacteria can fix N at moisture levels as low as 6% and in one study reached maximum N fixation rates at soil moistures between 22 and 42% (Jones 1977), though it did not specify whether this was in sandy or clayey soil. As soil moisture in Paracou always remained between 15 and 44% (Table S1) it is likely that diazotrophs had sufficient moisture to keep oxygen levels low and continue N fixation.

At Nouragues, the effect of soil moisture on FLNF rates was high (40% of variability explained by soil moisture alone, Table 2a) and seasonality caused an interesting pattern in the distribution of very low FLNF rate measurements (below detection limit). The vast majority of below detection limit measurements occurred during the dry season (Fig. S4 B) and when analysing only data for which FLNF rates were above the detection limit, we found a significant season effect in Nouragues with quantifiable rates (i.e. in samples above the detection limit) actually being higher in the dry season than in the wet season (Fig. S5 B). This meant that the range of FLNF rates was broader during the dry season than during the wet season (Fig. S5 A), indicating that N fixation hotspots were of increasing importance. These are typically found in tropical ecosystems (Pajares and Bohannan 2016) and reflect the very small-scale spatial heterogeneity of abiotic factors affecting the dynamics of the diazotroph community (Reed et al. 2010). As the soil dried out at the onset of the dry season the heterogeneity of water-filled pore space increased, leading to the creation of aerobic and anaerobic, as well as dry and mesic microsites that co-occured on a small spatial scale (Sexstone et al. 1985).

Besides water content, available P and pH were the most important predictors for soil FLNF at our sites. The pH in our soils is very low, ranging between 3.8 and 4.2 (Table S1). This is a relatively small range, yet pH has been identified to affect bacterial community composition and diversity (Rousk et al. 2010; Tripathi et al. 2014) or soil CO_2_ effluxes (Courtois et al. 2018) at the local scale even when changes are small. We were unable to find studies assessing the effect of pH on FLNF in tropical ecosystems, but nitrogenase activity of soil diazotrophs has been shown to increase with increased pH in a German pine forest situated in north-east Bavaria (Limmer and Drake 1996). Roper and Smith (1991) found that nitrogenase activity of bacteria extracted from clayey Australian soils reached its peak around pH 7.5, matching the nitrogenase pH optimum found by Pham and Burgess (1993), and decreased in more acidic soils. Their study, however, was carried out on soils that at the start of the experiment were only slightly acidic after which the pH was decreased during the course of the study. Their result is therefore not necessarily representative for N fixing microbial communities that developed in acidic soils, such as in our study. However, taking into account an enzymatic pH optimum of 7.5 (Pham and Burgess 1993) and decreased nitrogenase activity in more acidic temperate soils (Zahran 1999), a positive relationship between soil pH and FLNF rates in our acidic tropical forest sites is plausible.

Phosphorus can play a pivotal role in regulating N fixation rates and is often limiting the rate of this process in highly weathered lowland tropical soils (e. g. Camenzind et al. 2018; Reed et al. 2011). In Paracou, both P predictors were positively correlated with the FLNF rates, suggesting a higher activity of N fixing microbes when more P is present in the soil. Additions of P to the forest floor have shown to increase both diversity and abundance of diazotrophs in tropical soils (Reed et al. 2010), as well as increases in FLNF rates (e. g. Benner et al. 2007; Reed et al. 2013). The positive correlation between available P and total P on the one hand, and FLNF rates on the other, is in line with the longstanding idea that increased P benefits FLNF rates in tropical lowland forests. In addition to P, the model for Paracou also identified available N as a predictor, indicating that higher available N is linked to decreased FLNF rates. This is in line with what was observed in previous tropical forest studies, where additions of N led to the decrease of N fixation rates (e. g. Barron et al. 2009; Crews et al. 2000; Cusack et al. 2009). This observation is generally attributed to the theory that many heterotrophic N_2_ fixers are facultative fixers and able to down-regulate their fixation pathway when other sources of N are available and more cost-efficient to acquire (Menge et al. 2009). The topographic effect on FLNF rates in Paracou, effectively resulting in lower rates on the top than on bottom and slope plots is likely caused by the interplay of P and N in the Paracou soils as they also varied with topographic position. Total P was highest on the slope and available P highest on the bottom, while both were lowest on the top. Because of the higher clay content of the Paracou slope soils there are likely more aluminium and iron oxides (Tiessen et al. 1994) that are able to occlude more P, resulting in higher soil total P contents as seen on the slope. On the more sandy bottom landscape position metal oxides that can occlude P are likely scarcer and the higher water content, especially during rain events when runoff causes disproportionate changes in water status, leads to reduction of iron oxides (Colombo et al. 2014), liberating occluded P and provoking higher concentrations of available P.

In Nouragues, we found that water content, C and N performed equally well, each individually explaining 40% of the observed variation in FLNF rates, suggesting that the absolute concentrations of C and N in the soil explain a substantial part of the variation in FLNF rates. It is surprising that the model identified a positive effect of N content on FLNF rates, given that most studies associate increased N with decreased or unchanged fixation rates (Camenzind et al. 2018). Additionally, we would expect that available N, which was also included as potential variable but was not selected by the model, would be identified as a variable affecting FLNF rates instead of N content because no decomposition is needed before assimilation. Likely, the identification of N by the model is purely mathematical and caused by its near perfect correlation with C content (Table S3 B). The positive relationship of C with FLNF rates, which is predominantly carried out by heterotrophic diazotrophs (Sprent and Sprent 1990), is the result of decomposition of organic material and subsequent assimilation of additional C providing the energy necessary to carry out fixation. In Nouragues, the high correlation between C content and moisture (Table S3 B) partly explains their equal importance in the model. Their correlation might be due to the influence of soil moisture on decomposition rates, especially when it is very wet (negative relation) or very dry (positive relation). At high moisture levels, soil organic carbon and nitrogen stocks increase because of the slower decomposition in water-saturated soil (Van Sundert et al. 2018). Moreover, soils in Nouragues are clay-rich, exacerbating this effect as soils containing more clay stabilize and store more soil organic matter than sandy soils (Reis et al. 2014), such as those in Paracou. Both increased water and C can be beneficial for free-living diazotrophs as many species possess heterotrophic anaerobic metabolisms (Dixon and Kahn 2004) and proliferate under oxygen poor and carbon rich conditions.

In Nouragues the topographical patterns of N and P were different from Paracou, with much higher total P concentrations that occurred on the top landscape position instead of on the slopes, and much smaller differences in available P concentrations among the topographic positions. This likely played a role in the regulation of soil FLNF rate, causing them to remain high on the top landscape position. Because of this strong difference in topography effects between Nouragues and Paracou, we cannot draw general conclusions about landscape-scale variations in soil FLNF rates across topographies in French Guianese tropical forests. Instead, our results support the idea that soil FLNF rates at our sites varied in function of water and nutrient availability, similar to what was reviewed by Dynarski et al. (2018).

Lastly, it is interesting to note that total or available Mo was never selected as explanatory variable for soil FLNF rates, in spite of playing a regulatory role in FLNF at other sites (Barron et al. 2009; Reed et al. 2013; Wurzburger et al. 2012). Molybdenum concentrations in our study sites were slightly higher than those found elsewhere (Gupta and Lipsett 1981), and available Mo was ten times higher than in Panama (Wurzburger et al. 2012), which was the only study reporting the effect of available molybdenum on FLNF. Likely, the concentration of available Mo at our sites is high enough to preclude any regulatory role of Mo in this P-limited environment.

### Leaf litter free-living N_2_ fixation

Overall, leaf litter FLNF rates did not vary with topography and were instead driven mainly by water content and the N:P ratio of the leaf litter, as shown by the overall model explaining 46% of the variation (Table 2b). The negative influence of N:P ratio is in line with what was postulated by Reed et al. (2007) and likely illustrates a stoichiometric and energetic balance shift. In a decomposing leaf a decreasing N:P ratio leads to P becoming comparatively more abundant, shifting the stoichiometric balance to move away from P limitation to N limitation, favouring diazotrophs. Additionally, increased P in the environment may alleviate energetic constraints (see above) and enable diazotrophs to invest the required energy into fixing N. As the general model included data from both seasons, a large influence of water content, as evidenced by the largest beta value (Table 2b), was to be expected because litter FLNF rates were significantly affected by seasonality; wet season FLNF rates were up to nearly two times higher than dry season FLNF rates (Table 1) and the number of samples for which FLNF rates were below the detection limit was much smaller in the wet than in the dry season (Fig. S4 C & D). As expected, litter dried out severely during the dry season and water content decreased from an average ~64 to ~40% across both sites. Given that diazotrophs grow and proliferate in aqueous environments (Scott 1957), it is likely that leaf litter water content in the dry season dropped below a threshold of minimum water required for diazotroph proliferation, resulting in a net drop of FLNF rate. This response of diazotroph activity to leaf litter moisture was already observed in a sub-tropical karst forest, where researchers found that decreases in leaf litter moisture resulted in decreased FLNF rates (Li et al. 2018). The large beta value (2.63, Table 2b) associated with water content in the dry season model hints towards the disruptive effect of water shortage. This model explained 67% of the variation in litter FLNF rates and included only water content and litter N, which, just as the N:P ratio in the general model, had a negative effect on the FLNF rates. As mentioned earlier, N assimilation is cheaper than N fixation from an energetic point of view and when more N is present fixation will likely be down regulated (Menge et al. 2009). On the top landscape position in Paracou, also during the dry season, leaf litter water content was lowest while N:P ratio was highest. This likely led to a complete collapse of N fixation (Fig. 1c), because N was in ample supply compared to P and water was scarce. During the wet season, litter stoichiometry explained 33% of the variation in FLNF rates which likely reflects, at least partially, the interactions between seasonal changes in labile C availability, P content and N demand (Reed et al. 2007). Litterfall peaks at the onset of the dry season (Chave et al. 2010; Wagner et al. 2013) and once the wet season starts the daily rainfall provides a vehicle for the movement of readily decomposable, dissolved organic C (DOC) within the litter layer (Courtois et al. 2018). The N_2_-fixing microbial community in the litter layer is dominated by heterotrophic microorganisms (Sprent and Sprent 1990) and the influx of litter DOC provides an easily accessible energy source for these diazotrophs. Additionally, in our study, we found that the concentration of both P and N in leaf litter was higher in the wet season than in the dry season, similar to what was found in another study in Costa Rica (Wood et al. 2005). However, relative to N content, P content increased more towards the wet season, resulting in a lower N:P ratio during the wet season. In combination with more labile C input, this could stimulate N fixation through the relief of energetic constraints and the added advantage of being able to fix N compared to assimilating N from an environment where it is, comparatively, less abundant than in the dry season. This process was already observed in tallgrass prairie soils (Eisele et al. 1989).

In contrast to litter P content, which changed significantly across seasons yet showed no significant change along the topographical gradient, litter Mo content changed significantly between the two seasons and along the topographical gradient. Just as in soils, however, and in spite of its importance for N fixation (Kaiser et al. 2005; Seefeldt et al. 2009), we found no evidence to support a regulatory role for Mo content. Similar as in soils, litter Mo concentrations were ten-fold higher than those reported from other tropical sites (Barron et al. 2009; Bowell and Ansah 1993; Reed et al. 2013; Wurzburger et al. 2012) and likely too high to render a regulatory role to Mo in litter FLNF rates.

Lastly, in both soils and leaf litter our models were unable to explain more than 67% of the observed variation, and in most cases only around 40%. This means that often the majority of variation in FLNF rates could not be explained by the variables we selected and thus it is very likely that other factors not measured in this study contribute to the regulation of FLNF in our tropical lowland forests. Knowing the diazotroph community composition, in both soil and leaf litter, could enhance our understanding of their nutritional and environmental needs and help us estimate at what point parameters such as moisture, pH and P or N availability are beneficial or, inversely, detrimental. The likelihood that within a single diazotroph community both aerobic and anaerobic lifestyles can occur (Dixon and Kahn 2004) and that N fixing Archaea within the community may possess different nutritional requirements than bacterial diazotrophs (Leigh 2000) are additional reasons to study the diazotroph community composition. Also, we did not assess the iron (Fe) or the vanadium (V) availabilities at our sites while they have the potential of participating in FLNF rate regulation (Zhang et al. 2016). While Mo is a necessary co-factor of most nitrogenase enzymes (Igarashi and Seefeldt 2003), Fe is found in all known nitrogenases and the occurrence of ‘iron-only’ (Fe-Fe) nitrogenases has been widely documented (e. g. Yang et al. 2014; Zheng et al. 2018). The role of V in regulation of FLNF is understudied, but it is certain that the occurrence of an alternative enzymatic co-factor, namely the vanadium-iron (V-Fe) cofactor, is widespread and V availability may therefore also play a regulatory role in nitrogenase biosynthesis (Hu et al. 2012). A follow-up study investigating the community composition of the diazotrophs, specifically looking into the prevalence of aerobic and anaerobic organisms, combined with research into the occurrence of V-Fe nitrogenases and V and Fe availabilities in the soil and leaf litter could enable us to explain more of the observed variation than we presently could.

## Conclusion

Our study has shed light on the drivers behind FLNF in tropical soil and leaf litter on the Guiana Shield and has shown that the rates of FLNF are much lower than those estimated for most tropical forests elsewhere. Water, N and P played the main roles in determining FLNF rates in both sample types, while pH only regulated in the soil. The effect of seasonality differed between sample type and differences in FLNF regulation between sites could be observed in soils, but not in litter. Despite having been shown to influence N fixation rates in other mature tropical forests, the micro nutrient Mo played no role in the regulation of FLNF rates at our sites in French Guiana.

In the sandy site, Paracou, the stimulating effect of P and the inhibiting effect of N were the main drivers behind soil FLNF, but in the clayey site, Nouragues, soil FLNF was mainly stimulated by water or C content. Our models for soil FLNF could not explain more than 40% of the observed variation, illustrating the complexity of predicting fixation rates upon measured variables in an environment that is highly heterogeneous on a regional, local and even micro scale. In leaf litter we also identified water, N and P as main drivers, but the underlying mechanisms that caused variation may have been different compared to the soil. In the leaf litter we observed no differences in FLNF rates between sites, but during the dry season litter rates exhibited a drastic decline that was mainly related to water insufficiency and the inhibiting effect of N. During the wet season water was still of importance, but now stimulating effects of C and P also came into play. It is important to note that our litter wet season model explained only about 30% of the observed variation.

It is likely that in both soil and leaf litter diazotroph community composition and iron or vanadium availability had an influence on FLNF rates making them interesting to measure in future studies. In the larger framework of global change, where N deposition is expected to increase (Penuelas et al. 2013), P deposition to tropical forests may change (Gross et al. 2016) and the possibility of a drier Amazon basin (IPCC 2013) may cause disruptive changes to the FLNF rates in soil and litter. Nutrient addition studies may offer clues as to the response of FLNF to changes in N or P supply, but testing the influence of climatic changes in situ calls for a very specific type of studies.

## Electronic supplementary material

ESM 1 (DOCX 366 kb)
