# Supplementary material for: Regulation of nitrogen fixation from free-living organisms in soil and leaf litter of two tropical forests of the Guiana shield
Source: Plant Soil. 2019 Apr 1;450(1):93–110. doi: 10.1007/s11104-019-04012-1 (PMC7319290; doi:10.1007/s11104-019-04012-1)
Supplement: Supplementary file 1 — (DOCX 366 kb) [file 11104_2019_4012_MOESM1_ESM.docx]

**Supplementary information**

Table S1. Soil water content, stoichiometry and nutrient availabilities for both field sites and seasons on the three topographies

|  | **Paracou** | | | | | | |  | **Nouragues** | | | | | | |
| --- | --- | --- | --- | --- | --- | --- | --- | --- | --- | --- | --- | --- | --- | --- | --- |
|  | **Wet season** | | |  | **Dry season** | | |  | **Wet season** | | |  | **Dry season** | | |
|  | **Bottom** | **Slope** | **Top** |  | **Bottom** | **Slope** | **Top** |  | **Bottom** | **Slope** | **Top** |  | **Bottom** | **Slope** | **Top** |
| Gravimetric water (%) | 37 (2)^ab^ | 44 (2)^a^ | 36 (1)^b^ |  | 19 (1)^cd^ | 22 (1)^c^ | 15 (1)^d^ |  | 48 (5)^b^ | 55 (3)^b^ | 66 (3)^a^ |  | 40 (5)^c^ | 30 (1)^c^ | 52 (3)^b^ |
| C:N ratio | 17.8 (0.7)^b^ | 20.1 (1.1)^ab^ | 13 (0.8)^c^ |  | 18.7 (1.2)^ab^ | 18.8 (0.7)^ab^ | 21.0 (0.8)^a^ |  | 15.8 (0.3)^a^ | 17.5 (0.3)^a^ | 16.0 (0.5)^a^ |  | 16.3 (0.6)^a^ | 16.0 (0.3)^a^ | 16.3 (0.4)^a^ |
| Total C (%) | 3.1 (0.3)^b^ | 4.9 (0.5)^ab^ | 3.3 (0.3)^b^ |  | 3.6 (0.7)^b^ | 4.8 (0.8)^ab^ | 5.9 (0.8)^a^ |  | 5.0 (0.7)^a^ | 5.8 (0.4)^a^ | 6.6 (0.4)^a^ |  | 4.6 (0.8)^a^ | 4.1 (0.2)^a^ | 8.2 (0.9)^a^ |
| Total N (%) | 0.17 (0.01)^b^ | 0.24 (0.01)^a^ | 0.26 (0.01)^a^ |  | 0.18 (0.03)^b^ | 0.24 (0.02)^a^ | 0.27 (0.03)^a^ |  | 0.32 (0.04)^b^ | 0.33 (0.02)^b^ | 0.42 (0.02)^a^ |  | 0.27 (0.04)^b^ | 0.25 (0.01)^b^ | 0.49 (0.04)^a^ |
| Total P  (mg kg^-1^) | 78 (3)^bc^ | 112 (3)^a^ | 65 (3)^ce^ |  | 63 (3)^de^ | 96 (4)^b^ | 58 (4)^d^ |  | 85 (8)^cd^ | 93 (4)^c^ | 272 (5)^a^ |  | 69 (8)^cd^ | 75 (3)^d^ | 267 (6)^b^ |
| Total Mo  (mg kg^-1^) | 10.9 (1.1)^a^ | 6.6 (0.7)^b^ | 10.3 (1)^a^ |  | 2.0 (0.1)^c^ | 1.4 (0.1)^c^ | 1.8 (0.1)^c^ |  | 6.3 (1.1)^a^ | 5.6 (0.6)^a^ | 1.5 (0.2)^b^ |  | 5.8 (0.7)^a^ | 4.7 (0.6)a | 0.1 (0)^b^ |
| Available N  (mg kg^-1^) | 10.4 (0.8)^b^ | 24.7 (1.9)^a^ | 15.3 (1.4)^b^ |  | 4.6 (0.3)^c^ | 11.4 (0.7)^b^ | 5.9 (0.3)^c^ |  | 9.4 (0.9)^bc^ | 7.7 (0.3)^c^ | 16.4 (0.9)^a^ |  | 9.0 (0.6)^bc^ | 11.0 (1.0)^b^ | 15.0 (1.0)^a^ |
| Available P  (mg kg^-1^) | 3.3 (0.3)^a^ | 1.3 (0.1)^c^ | 1.3 (0.1)^cd^ |  | 2.7 (0.2)^b^ | 0.9 (0.1)^d^ | 1.2 (0.1)^cd^ |  | 1.8 (0.2)^b^ | 0.9 (0)^df^ | 1.0 (0)^ef^ |  | 2.0 (0.1)^a^ | 1.2 (0.1)^ce^ | 1.3 (0.1)^cd^ |
| Available Mo  (µg kg^-1^) | 6.42 (0.9)^b^ | 8.53 (1.61)^a^ | 3.96 (0.65)^bc^ |  | 3.97 (0.76)^c^ | 5.97 (0.83)^b^ | 3.52 (0.31)^c^ |  | 3.47 (0.34)^a^ | 1.49 (0.42)^bc^ | 0.36 (0.17)^c^ |  | 0.61 (0.26)^c^ | 2.48 (0.34)^ab^ | 3.76 (1.18)^a^ |
| pH | 4.21 (0.03)^a^ | 4.04 (0.02)^b^ | 4.06 (0.01)^b^ |  | 3.99 (0.02)^b^ | 4.00 (0.02)^b^ | 3.88 (0.02)^c^ |  | 3.95 (0.02)^a^ | 3.81 (0.01)^bc^ | 3.78 (0.06)^c^ |  | 3.82 (0.03)^bc^ | 3.80 (0.02)^c^ | 3.90 (0.02)^ab^ |
| Clay (%) | 8.5 (0.5)^b^ | 17.5 (0.9)^a^ | 9.3 (0.1)^b^ |  | 8.7 (0.5)^b^ | 17.5 (0.9)^a^ | 9.3 (0.1)^b^ |  | 18.2 (0.5)^c^ | 26.0 (1.4)^b^ | 42.8 (0.9)^a^ |  | 18.5 (0.6)^c^ | 25.7 (1.4)^b^ | 42.4 (0.8)^a^ |
| Sand (%) | 77.3 (0.9)^a^ | 63.5 (1.4)^b^ | 76.2 (0.2)^a^ |  | 76.6 (0.9)^a^ | 63.5 (1.4)^b^ | 76.2 (0.2)^a^ |  | 63.8 (0.9)^a^ | 53.2 (2.6)^b^ | 22.8 (2.0)^c^ |  | 63.4 (1.0)^a^ | 53.5 (2.6)^b^ | 23.2 (2.0)^c^ |
| Bulk density  (kg m^-2^) | 58.0 (1.9)^a^ | 51.9 (1.4)^b^ | 56.4 (2.5)^a^ |  | 58.1 (1.8)^a^ | 51.9 (1.4)^b^ | 56.4 (2.5)^a^ |  | 47.1 (2)^a^ | 45.9 (1.2)^a^ | 39.1 (1.3)^b^ |  | 46.4 (2.2)^a^ | 45.9 (1.2)^a^ | 40.0 (1.4)^b^ |

Values are means with standard errors in parentheses. Letters denote significant differences (linear mixed effects model with Season and Topography as factors, followed by post hoc tests and with p<0.05 as significance level) within a site.

Table S2. Leaf litter water content and stoichiometry at Paracou and Nouragues for both seasons and on the three topographies.

|  | **Paracou** | | | | | | |  | **Nouragues** | | | | | | |
| --- | --- | --- | --- | --- | --- | --- | --- | --- | --- | --- | --- | --- | --- | --- | --- |
|  | **Wet season** | | |  | **Dry season** | | |  | **Wet season** | | |  | **Dry season** | | |
|  | **Bottom** | **Slope** | **Top** |  | **Bottom** | **Slope** | **Top** |  | **Bottom** | **Slope** | **Top** |  | **Bottom** | **Slope** | **Top** |
| Gravimetric water (%) | 0.64 (0.01)^a^ | 0.67 (0.01)^a^ | 0.58 (0.02)^b^ |  | 0.42 (0.02)^c^ | 0.31 (0.01)^d^ | 0.22 (0.01)^e^ |  | 0.67 (0.01)^a^ | 0.66 (0.01)^ab^ | 0.62 (0.01)^b^ |  | 0.60 (0.01)^b^ | 0.44 (0.01)^c^ | 0.42 (0.03)^c^ |
| C:N ratio | 36.1 (0.9)^b^ | 34.9 (0.7)^b^ | 37.5 (1)^b^ |  | 42 (1.5)^a^ | 39.5 (0.9)^a^ | 42.1 (1.2)^a^ |  | 31.5 (1.8)^b^ | 34.2 (0.8)^b^ | 34.7 (0.9)^b^ |  | 33.7 (1.2)^b^ | 40.8 (1.6)^a^ | 38.9 (1.4)^a^ |
| N:P ratio | 59.8 (2.2)^c^ | 60.2 (3.3)^c^ | 74.6 (3.1)^b^ |  | 63.0 (3.7)^b^ | 74.9 (3.7)^ab^ | 83.6 (5.5)^a^ |  | 60.4 (3.1)^b^ | 64.6 (4.0)^b^ | 58.7 (3.1)^b^ |  | 82.3 (5.2)^a^ | 79.6 (3.9)^a^ | 70.8 (2.9)^a^ |
| Total C (%) | 40.5 (1.1)^b^ | 43.9 (0.7)^b^ | 44.6 (1.3)^b^ |  | 45.4 (0.6)^a^ | 46.0 (0.3)^a^ | 45.1 (0.6)^a^ |  | 41.6 (1.1)^b^ | 45 (0.6)^a^ | 45.6 (0.4)^a^ |  | 45.1 (0.4)^a^ | 47.0 (0.7)^a^ | 45.9 (0.5)^a^ |
| Total N (%) | 1.14 (0.04)^a^ | 1.28 (0.03)^a^ | 1.21 (0.05)^a^ |  | 1.11 (0.05)^b^ | 1.19 (0.03)^b^ | 1.09 (0.03)^b^ |  | 1.39 (0.08)^a^ | 1.33 (0.03)^a^ | 1.34 (0.04)^a^ |  | 1.37 (0.06)^b^ | 1.19 (0.04)^b^ | 1.2 (0.03)^b^ |
| Total P (mg kg^-1^) | 200 (14)^a^ | 227 (13)^a^ | 171 (14)^a^ |  | 190 (24)^b^ | 171 (10)^b^ | 140 (11)^b^ |  | 251 (26)^a^ | 220 (16)^a^ | 240 (14)^a^ |  | 180 (12)^b^ | 157 (9)^b^ | 176 (9)^b^ |
| Total Mo (mg kg^-1^) | 2.8 (0.3)^b^ | 1.5 (0.2)^c^ | 1.8 (0.4)^c^ |  | 3.4 (0.7)^a^ | 1.9 (0.2)^ab^ | 3.8 (0.8)^ab^ |  | 1.7 (0.3)^a^ | 0.9 (0.2)^b^ | 0.5 (0.1)^b^ |  | 1.8 (0.4)^a^ | 0.7 (0.1)^b^ | 0.7 (0.1)^b^ |
| Bulk density (g m^-2^) | 468.2 (62.5)^a^ | 370.4 (23.2)^b^ | 469.1 (18.9)^a^ |  | 367.7 (23.0)^b^ | 340.2 (20.7)^c^ | 347.6 (12.8)^bc^ |  | 441.0 (29.7)^b^ | 380.7 (20.7)^b^ | 336.7 (20.5)^b^ |  | 571.8 (94.2)^a^ | 547.5 (58.4)^a^ | 682.3 (72.6)^a^ |

Values are means with standard errors in parentheses. Letters denote significant differences (linear mixed effects model with Season and Topography as factors, followed by post hoc tests and with p<0.05 as significance level) within a site.

Table S3 Correlation matrix showing Pearson’s r for the variable used in the stepwise regression analysis. Data from A Paracou soil, B Nouragues soil and C leaf litter from both sites. Data was averaged per plot prior to calculation. Abbreviations: C = total C, N = total N, P = total P, Mo = total Mo, C:N = C:N ratio, N:P = N:P ratio, P_in_ = available P, N_in_ = available N, Mo_in_ = available Mo, Moisture = water content, pH = pH, Clay = % clay, Sand = % sand and BD = bulk density

| **A** | | | | | | | | | | | | |
| --- | --- | --- | --- | --- | --- | --- | --- | --- | --- | --- | --- | --- |
| Soil variables | C | N | C:N | P | Mo | N_in_ | P_in_ | Mo_in_ | pH | Clay | Sand | BD |
| Moisture | -0,16 | 0,06 | -0,24 | 0,45 | 0,64 | 0,68 | 0,05 | 0,55 | 0,55 | 0,31 | -0,33 | -0,23 |
| C |  | 0,78 | 0,63 | 0,03 | -0,48 | 0,01 | -0,37 | -0,06 | -0,43 | 0,20 | -0,20 | -0,09 |
| N |  |  | 0,04 | 0,04 | -0,18 | 0,12 | -0,51 | -0,14 | -0,39 | 0,17 | -0,18 | -0,23 |
| C:N |  |  |  | 0,02 | -0,52 | -0,07 | -0,01 | 0,12 | -0,19 | 0,11 | -0,12 | 0,04 |
| P |  |  |  |  | -0,06 | 0,69 | -0,26 | 0,49 | 0,00 | 0,85 | -0,83 | -0,63 |
| Mo |  |  |  |  |  | 0,26 | 0,34 | 0,30 | 0,69 | -0,31 | 0,31 | 0,21 |
| N_in_ |  |  |  |  |  |  | -0,25 | 0,33 | 0,09 | 0,64 | -0,63 | -0,45 |
| P_in_ |  |  |  |  |  |  |  | 0,02 | 0,36 | -0,51 | 0,53 | 0,54 |
| Mo_in_ |  |  |  |  |  |  |  |  | 0,52 | 0,45 | -0,44 | -0,22 |
| pH |  |  |  |  |  |  |  |  |  | -0,14 | 0,09 | 0,18 |
| Clay |  |  |  |  |  |  |  |  |  |  | -0,99 | -0,68 |
| Sand |  |  |  |  |  |  |  |  |  |  |  | 0,73 |

| **B** | | | | | | | | | | | | |
| --- | --- | --- | --- | --- | --- | --- | --- | --- | --- | --- | --- | --- |
| Soil variables | C | N | C:N | P | Mo | N_in_ | P_in_ | Mo_in_ | pH | Clay | Sand | BD |
| Moisture | 0,75 | 0,77 | 0,14 | 0,65 | -0,45 | 0,48 | -0,36 | -0,21 | -0,20 | 0,59 | -0,62 | -0,64 |
| C |  | 0,97 | 0,25 | 0,67 | -0,52 | 0,50 | -0,18 | 0,22 | -0,12 | 0,61 | -0,63 | -0,63 |
| N |  |  | 0,05 | 0,75 | -0,63 | 0,62 | -0,23 | 0,15 | -0,11 | 0,70 | -0,72 | -0,74 |
| C:N |  |  |  | -0,11 | 0,37 | -0,36 | -0,02 | 0,13 | -0,05 | -0,18 | 0,20 | 0,34 |
| P |  |  |  |  | -0,70 | 0,80 | -0,48 | -0,10 | 0,03 | 0,90 | -0,93 | -0,79 |
| Mo |  |  |  |  |  | -0,70 | 0,40 | 0,06 | 0,19 | -0,86 | 0,87 | 0,87 |
| N_in_ |  |  |  |  |  |  | -0,28 | -0,05 | -0,25 | 0,81 | -0,82 | -0,71 |
| P_in_ |  |  |  |  |  |  |  | 0,23 | 0,11 | -0,63 | 0,60 | 0,54 |
| Mo_in_ |  |  |  |  |  |  |  |  | 0,36 | -0,12 | 0,14 | 0,11 |
| pH |  |  |  |  |  |  |  |  |  | -0,19 | 0,15 | 0,09 |
| Clay |  |  |  |  |  |  |  |  |  |  | -0,99 | -0,87 |
| Sand |  |  |  |  |  |  |  |  |  |  |  | 0,89 |

| **C** | | | | | | |
| --- | --- | --- | --- | --- | --- | --- |
| Litter variables | C | N | C:N | P | N:P | Mo |
| Moisture | -0,29 | 0,55 | -0,67 | 0,65 | -0,50 | -0,31 |
| C |  | 0,13 | 0,37 | -0,11 | 0,25 | -0,49 |
| N |  |  | -0,85 | 0,61 | -0,13 | -0,46 |
| C:N |  |  |  | -0,61 | 0,24 | 0,22 |
| P |  |  |  |  | -0,83 | -0,26 |
| N:P |  |  |  |  |  | 0,13 |

Table S4 Comparison of FLNF rates measured in different studies carried out in primary tropical rainforests. Rates for soil and leaf litter are given and expressed as nmol of ethylene produced per gram of substrate per hour (nmol g^-1^ h^-1^).

| **Substrate** | **Country** | **Location** | **FLNF Rate (nmol g^-1^ h^-1^)** | **Reference** |
| --- | --- | --- | --- | --- |
| Litter | Hawai | Pahoehoe | 2.5 (0.4) | Vitousek 1999 |
|  | Hawai | A’a | 4.0 (1.4) | Vitousek 1999 |
|  |  |  |  |  |
|  | Hawai | Thurston | 3.15 (0.86) | Crews 2000 |
|  | Hawai | Laupahoehoe | 1.25 (0.31) | Crews 2000 |
|  | Hawai | Kokee | 1.08 (0.27) | Crews 2000 |
|  |  |  |  |  |
|  | Hawai | Pahoehoe | 7.42 (1.85) | Vitousek 2000 |
|  | Hawai | Thurston | 8.38 (2.10) | Vitousek 2000 |
|  | Hawai | Laupahoehoe | 1.93 (0.48) | Vitousek 2000 |
|  | Hawai | Kokee | 3.22 (0.81) | Vitousek 2000 |
|  |  |  |  |  |
|  | Costa Rica | Osa Peninsula, Ultisol | 8.82 (5.50) | Reed et al 2007 |
|  | Costa Rica | Osa Peninsula, Mollisol | 5.89 (4.75) | Reed et al 2007 |
|  |  |  |  |  |
|  | Panama |  | 0.53 (0.17) | Barron et al. 2009 |
|  |  |  |  |  |
|  | Puerto Rico | Wet tropical rainforest | 2.0 (0.5) | Cusack 2009 |
|  | Puerto Rico | Lower montane rainforest | 1.2 (0.5) | Cusack 2009 |
|  |  |  |  |  |
|  | Costa Rica | Osa Peninsula | 11.39 (2.75) | Reed et al 2010 |
|  |  |  |  |  |
|  | Panama | Fairchild | 6.52 (1.00) | Wurzburger et al. 2012 |
|  | Panama | AVA | 0.34 (0.08) | Wurzburger et al. 2012 |
|  | Panama | Gigante | 0.38 (0.06) | Wurzburger et al. 2012 |
|  | Panama | Barro Verde | 1.84 (0.42) | Wurzburger et al. 2012 |
|  | Panama | Zetek | 0.48 (0.21) | Wurzburger et al. 2012 |
|  | Panama | Rio Paja | 1.58 (0.23) | Wurzburger et al. 2012 |
|  |  |  |  |  |
|  | Costa Rica | Osa Peninsula | 3.77 (0.46) | Reed et al. 2013 |
|  |  |  |  |  |
|  | Costa Rica | Osa Peninsula | 0.60 (0.15) | Sullivan et al. 2014* |
|  |  |  |  |  |
|  | French Guiana | Paracou | 0.32 (0.10) | This study |
|  | French Guiana | Nouragues | 0.18 (0.06) | This study |
|  |  |  |  |  |
| Soil | Costa Rica | Osa Peninsula, Ultisol | 0.080 (0.013) | Reed et al 2007 |
|  | Costa Rica | Osa Peninsula, Mollisol | 0.042 (0.009) | Reed et al 2007 |
|  |  |  |  |  |
|  | Puerto Rico | Wet tropical rainforest | 0.11 (0.03) | Cusack 2009 |
|  | Puerto Rico | Lower montane rainforest | 0.06 (0.02) | Cusack 2009 |
|  |  |  |  |  |
|  | Ecuador | 1000 m | 0.179 (0.112) | Matson et al. 2014 |
|  | Ecuador | 2000 m | 0.313 (0.156) | Matson et al. 2014 |
|  | Ecuador | 3000 m | 0.223 (0.134) | Matson et al. 2014 |
|  |  |  |  |  |
|  | Costa Rica | Osa Peninsula | 0.017 (0.004) | Sullivan et al. 2014* |
|  |  |  |  |  |
|  | French Guiana | Paracou | 0.011 (0.005) | This study |
|  | French Guiana | Nouragues | 0.021 (0.011) | This study |

* For this study we found no bulk density reported for soil and litter. To calculate the amount of ethylene produced from the kg N ha^-1^ y^-1^ reported in the study we used the bulk density values we measured in French Guiana.


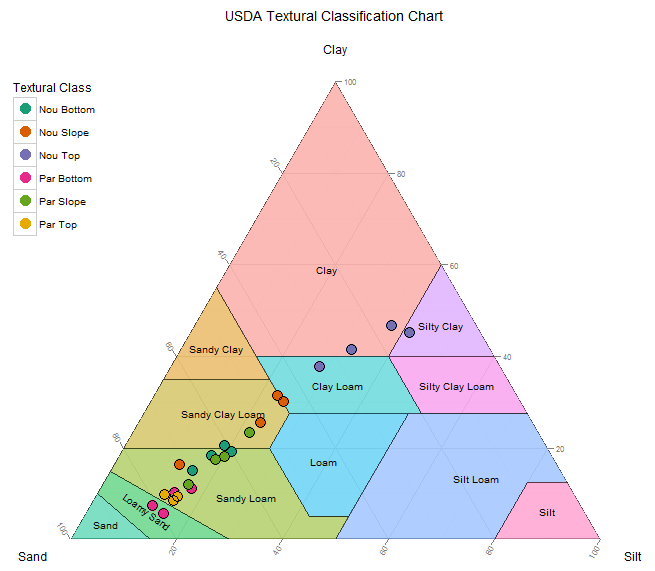


Figure S1. Soil classification based on texture for each of the twelve plots in Paracou and Nouragues. Dots are plot averages.


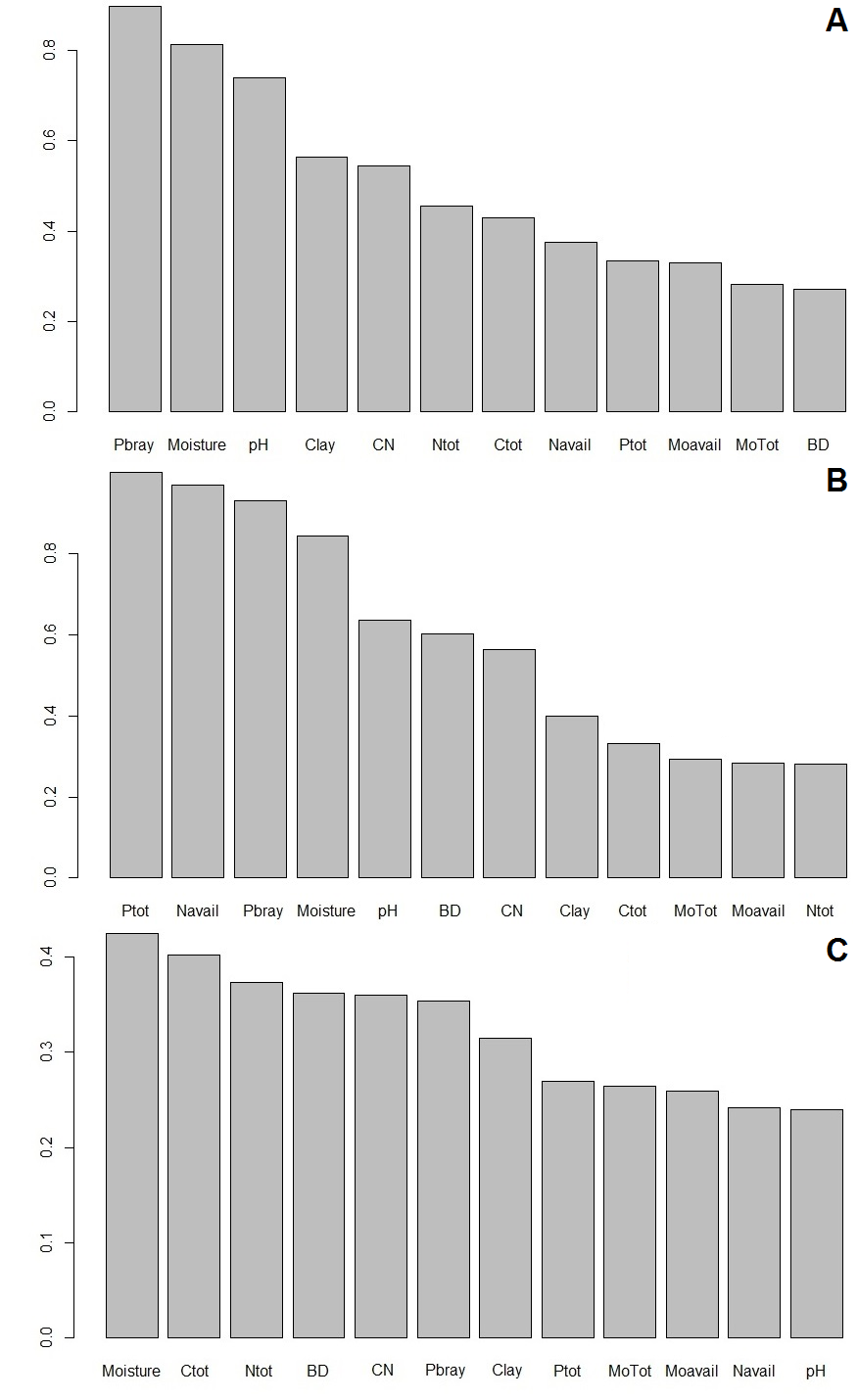


Figure S2 Relative importance of physico-chemical variables in the overall soil dataset (A), in Paracou (B) and in Nouragues (C). Higher relative importance means the predictor value is more likely to play a significant role in explaining the observed variation in FLNF rate (Burnham and Anderson 2002). Relative importance was calculated by summing the Akaike weights of each model, from all possible first order models, in which the variable participated. Moisture = water content, Ctot = total C, Ntot = total N, Ptot = total P, MoTot = total Mo, CN = C:N ratio, Navail = available N, Pbray = available P, Moavail = available Mo, pH = pH, Clay = percentage clay content and BD = bulk density.


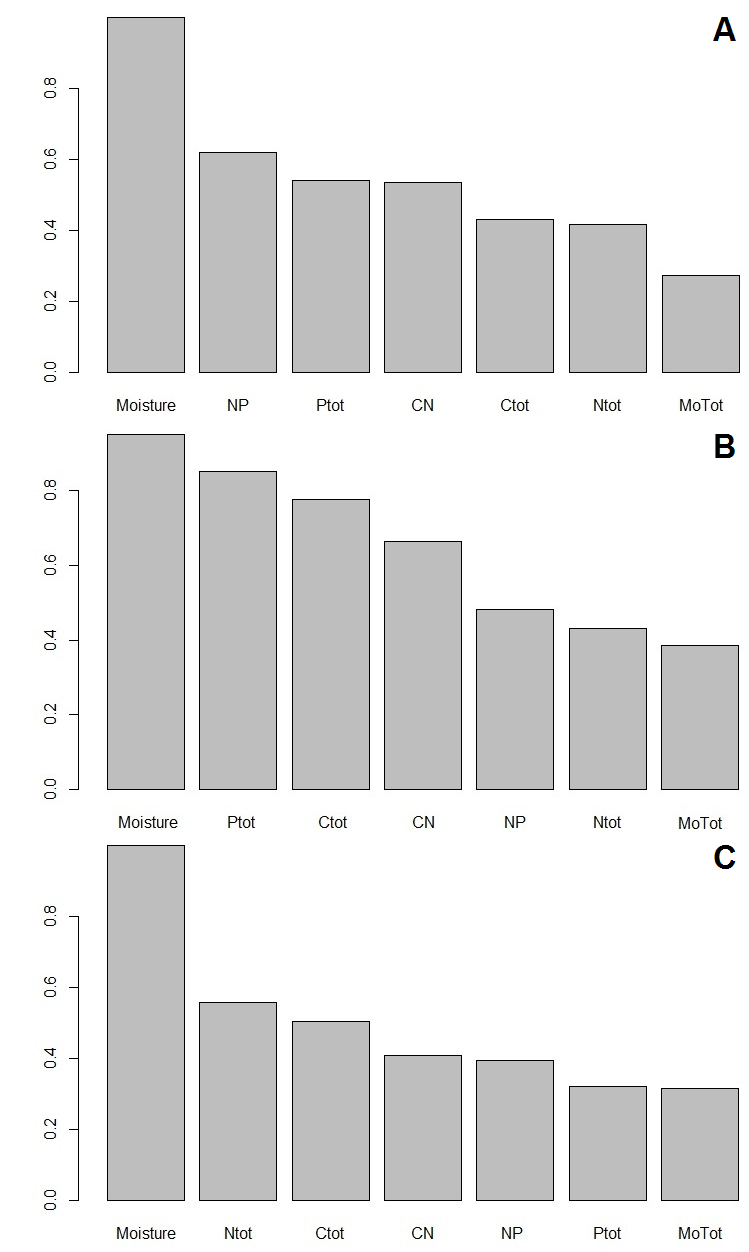


Figure S3 Relative importance of physico-chemical variables in the overall litter dataset (A), in the wet season (B) and in the dry season (C). Higher relative importance means the predictor value is more likely to play a significant role in explaining the observed variation in FLNF rate (Burnham and Anderson 2002). Relative importance was calculated by summing the Akaike weights of each model, from all possible first order models, in which the variable participated. Moisture = water content, Ctot = total C, Ntot = total N, Ptot = total P, MoTot = total Mo, CN = C:N ratio and NP = N:P ratio.


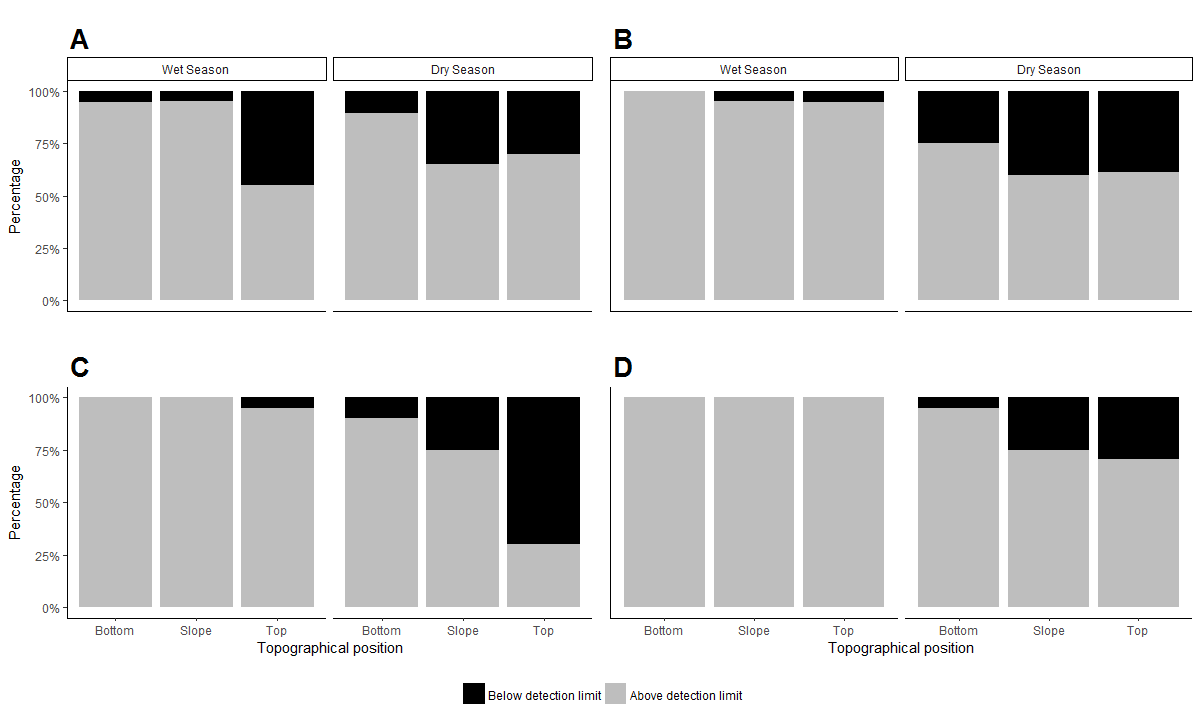


Figure S4. Percentage of N fixation rates below (black) and above (grey) the detection limit as a function of season and topography for (A) Paracou soil, (B) Nouragues soil, (C) Paracou leaf litter and (D) Nouragues leaf litter.


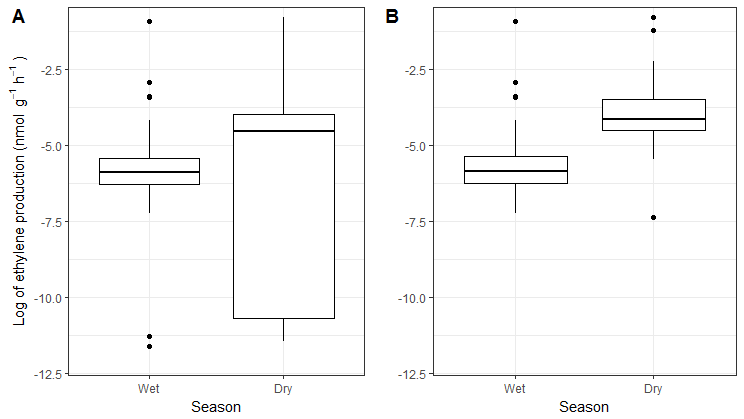


Figure S5. Boxplots comparing the effect of season on N fixation in Nouragues soils using the data set containing all datapoints (A) and the data set excluding datapoints below the detection limit (B).
